# AI-guided wound closure: complementing surgical judgment

**DOI:** 10.1097/MS9.0000000000004724

**Published:** 2026-01-22

**Authors:** Muhammad Khizar, Muhammad Zaib, Nukhbat-Ul Nisa, Hasiba Karimi, Hasibullah Aminpoor

**Affiliations:** a Faculty of Medicine, Georgian American University, Tbilisi, Georgia; b Faculty of Medicine, Shaikh Khalifa Bin Zayed Al Nahyan Medical and Dental College, Lahore, Pakistan; c Faculty of Medicine, Bezmialem Vakif University, Istanbul, Turkey; d Faculty of Medicine, Kabul University of Medical Sciences “Abu Ali Ibn Sina”, Kabul, Afghanistan

**Keywords:** artificial intelligence, robotic surgery, surgical wound closure

## Abstract

Artificial intelligence (AI) is redefining the precision of surgical wound closure through innovations in deep learning, computer vision, and robotic control. These technologies enable automated wound assessment, trajectory planning, and suturing assistance, with applications emerging across the United States, the United Kingdom, Japan, and India. Yet, the art of closure balancing tension, perfusion, and tissue integrity remains a fundamentally human skill. This work additionally highlights the current gap between theoretical AI capabilities and clinical validation, clarifies tissue-specific and interpretability challenges, and outlines concrete research pathways, including prospective trials, interpretable AI, and multimodal fusion systems. This letter discusses the clinical and technical aspects of AI-guided wound closure, emphasizing the importance of maintaining surgeon oversight. It argues that AI can enhance, but not supplant, human judgment, aligning with international guidelines on transparency and safety in AI deployment. Widespread clinical adoption will require rigorous validation, equitable access, and adherence to frameworks such as the TITAN Guidelines to ensure ethical and effective integration into surgical practice.

*To the Editor,*

Artificial intelligence (AI) has rapidly advanced into the surgical domain, reshaping concepts of precision and autonomy in operative care. Recent breakthroughs in large language models, protein structure prediction systems, and clinically oriented computer vision pipelines have accelerated interest in AI-enabled intraoperative decision support, underscoring a broader technological shift across medicine^[1,2]^. Among the most promising frontiers is AI-guided wound closure, where computer vision, deep learning, and robotic systems aim to standardize suturing and reduce human variability^[3]^. As global surgical workloads rise, such innovations could address disparities in access and outcomes by enhancing efficiency and accuracy across diverse healthcare settings^[4]^.

However, a central concern must be made explicit at the outset: there remains a substantial gap between the theoretical capabilities of AI-guided wound closure systems and the clinical validation required for their safe adoption^[5,6]^. This gap warrants critical, evidence-based evaluation rather than passive or overly optimistic promotion^[3]^.

Clinically, wound closure remains central to successful healing. Proper tissue apposition and tension balance preserve perfusion and prevent infection. Inadequate closure, conversely, increases rates of dehiscence and surgical site infection^[7]^. AI models have begun to assist in this domain by quantifying wound geometry, classifying tissue types, and suggesting closure parameters using convolutional neural networks and image-based learning^[7]^. However, the subtle intraoperative judgments of experienced surgeons, particularly in handling friable or contaminated tissue, remain beyond the reach of current algorithms.

Recent developments highlight the feasibility of autonomous or semi-autonomous closure. Ostrander *et al* demonstrated that robotic platforms can achieve near-human needle placement precision using deep neural trajectory mapping^[8]^. Experimental systems such as Touch Surgery (UK) and Medtronic’s Hugo™ (USA) employ integrated AI for motion tracking and suture planning. In Japan, collaborative robotics programs have piloted AI-assisted microsuturing, while Indian research teams explore cost-effective 3D wound assessment and predictive closure algorithms^[4]^. These international efforts indicate that AI can complement surgical expertise by providing standardization without compromising adaptability.

Despite progress, challenges persist. Several categories of data bias remain particularly relevant: (1) wound datasets disproportionately representing lighter skin tones, clean surgical fields, or specific geographic populations; (2) limited representation of complex wounds such as burns, traumatic lacerations, or contaminated incisions; and (3) inconsistent labeling standards across institutions. These biases directly affect algorithmic performance and generalizability^[9]^.

Cross-tissue validation is equally challenging because different tissues exhibit distinct mechanical properties, perfusion patterns, and responses to tension. A model trained predominantly on abdominal fascia, for example, may perform suboptimally when applied to delicate periorbital tissue. Without rigorous, tissue-specific validation, errors in suture spacing or tension prediction could compromise healing^[9]^.

Interpretability also remains a major barrier. Black-box tension predictions or trajectory recommendations reduce surgeon trust and limit real-world uptake. Interpretable AI tools such as visual heatmaps showing which wound regions influence tension estimation are essential for allowing surgeons to understand, verify, and override AI-generated decisions^[9]^.

Ethical integration requires transparent algorithmic design, rigorous prospective trials, and global accessibility strategies. As emphasized by emerging surgical governance frameworks, AI should augment, not replace, the surgeon’s judgment^[3]^. In the specific context of wound closure, AI is best suited for assisting with quantifiable, rule-based tasks such as suture spacing calculation, real-time tension prediction, suture trajectory planning, and automated documentation of closure quality. Conversely, surgeons must retain full responsibility for assessing tissue viability, managing contaminated or ischemic tissue, responding to unexpected bleeding or anatomical variations, and adapting closure strategy during intraoperative complications tasks requiring contextual reasoning, tactile feedback, and situational awareness that AI cannot yet replicate^[3,7,8]^. Our work is in line with the TITAN Guidelines on the need for transparency in AI use in healthcare^[10]^.

Future progress in this field will depend on three concrete research priorities: large-scale, multicenter, prospective clinical trials validating AI-guided closure systems across diverse surgical specialties and patient populations^[5,6,8]^; development of interpretable (XAI) systems that communicate their decision-making rationale through visual or quantitative cues^[7,9]^; and multimodal data fusion approaches that integrate computer vision with physiological signals such as tissue perfusion, oxygenation, or microvascular flow to provide comprehensive closure decision support^[7,8]^. These pathways provide feasible, clinically meaningful directions for translation.

## Data Availability

Not applicable.

## References

[1] Guo SB Liu DY Fang XJ. Current concerns and future directions of large language model chatGPT in medicine: a machine-learning-driven global-scale bibliometric analysis. Int J Surg 2025:10–97. doi:10.1097/JS9.0000000000003668 41133425

[2] Guo SB Meng Y Lin L. Artificial intelligence alphafold model for molecular biology and drug discovery: a machine-learning-driven informatics investigation. Mol Cancer 2024;23:223. 39369244 10.1186/s12943-024-02140-6 PMC11452995

[3] Loftus TJ Tighe PJ Filiberto AC. Artificial intelligence and surgical decision-making. JAMA Surg 2020;155:148–58. 31825465 10.1001/jamasurg.2019.4917 PMC7286802

[4] Ng JK Wah K. The rise of robotics and AI-assisted surgery in modern healthcare. J Robot Surg 2025;19:311. 40540146 10.1007/s11701-025-02485-0 PMC12181090

[5] Yu Z. Beyond hype: separating large language model-driven health literacy from clinical decision support system diagnostic rigour. Aust Crit Care 2025;38:101265. 40505386 10.1016/j.aucc.2025.101265

[6] Hashimoto DA Rosman G Rus D. Artificial intelligence in surgery: promises and perils. Ann Surg 2018;268:70–76. 29389679 10.1097/SLA.0000000000002693 PMC5995666

[7] Chen MY Cao MQ Xu TY. Progress in the application of artificial intelligence in skin wound assessment and prediction of healing time. Am J Transl Res 2024;16:2765–76. 39114681 10.62347/MYHE3488 PMC11301465

[8] Ostrander BT Mulier KE Fong ZV. The current state of autonomous suturing: a systematic review. Surg Endosc 2024;38:2383–97. 38553597 10.1007/s00464-024-10788-w

[9] Mittermaier M Raza MM Kvedar JC. Bias in AI-based models for medical applications: challenges and mitigation strategies. NPJ Digit Med 2023;6:113. 37311802 10.1038/s41746-023-00858-z PMC10264403

[10] Agha RA Mathew G Rashid R. Transparency In The reporting of Artificial INtelligence – the TITAN guideline. Prem J Sci 2025;10:100082.

